# Supplementary material for: Dynalign II: common secondary structure prediction for RNA homologs with domain insertions
Source: Nucleic Acids Res. 2014 Nov 21;42(22):13939–48. doi: 10.1093/nar/gku1172 (PMC4267632; doi:10.1093/nar/gku1172)
Supplement: SUPPLEMENTARY DATA [file supp_gku1172_nar-02021-z-2014-File012.zip › manual/RNA_class/html/hierarchy.html]

RNAstructure Classes: Hierarchical Index

- Main Page
- Classes
- Files

- Class List
- Class Hierarchy
- Class Members

# Class Hierarchy

This inheritance list is sorted roughly, but not completely, alphabetically:

- Multilign\_object
  - Multifind\_object
  - Thermodynamics
    - RNA
      - HybridRNA- Oligowalk\_object
    - TwoRNA
      - Dynalign\_object

---

Generated on Tue Jul 30 10:52:29 2013 for RNAstructure Classes by 
 1.5.7.1
